# Supplementary material for: Semi-synthetic nanobody-ligand conjugates exhibit tunable signaling properties and enhanced transcriptional outputs at neurokinin receptor-1
Source: bioRxiv. 2023 Oct 8:2023.10.08.561411. Preprint. [Version 1] doi: 10.1101/2023.10.08.561411 (PMC10659424; doi:10.1101/2023.10.08.561411)
Supplement: Supplement 1 [file media-1.pdf]

Supporting Information

Semi-synthetic nanobody-ligand conjugates exhibit tunable signaling properties and enhanced transcriptional outputs at neurokinin receptor-1

Nayara Braga Emidio[1], Ross W. Cheloha[1*]

[1] Laboratory of Bioorganic Chemistry, National Institute of Diabetes, Digestive, and Kidney Diseases, National Institutes of Health, Bethesda, Maryland 20894, United States.

* Corresponding author (ross.cheloha@nih.gov)

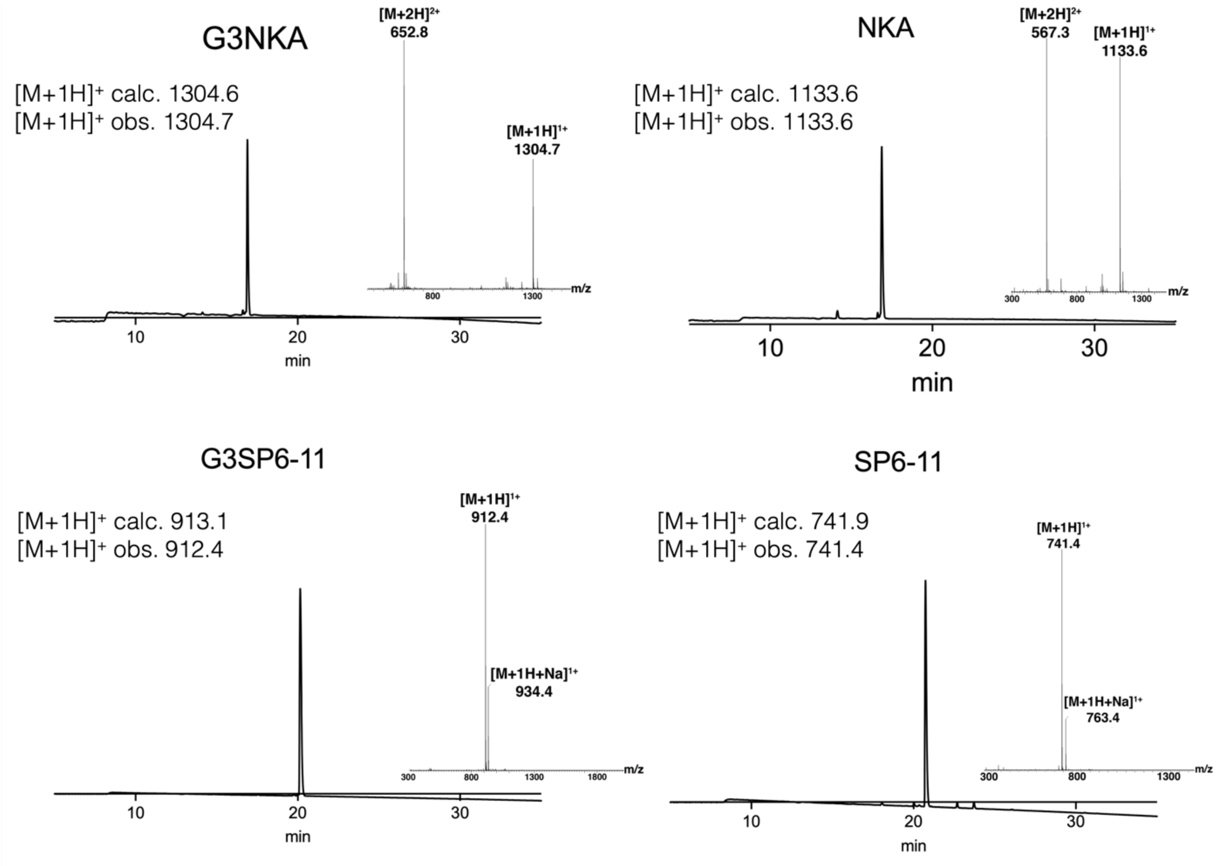

Figure S1. Analytical HPLC and high-resolution MS analysis of G3NKA, NKA, G3SP$_{6\text{-}11}$ and SP$_{6\text{-}11}$. HPLC and mass spectrometry were performed according to Methods.

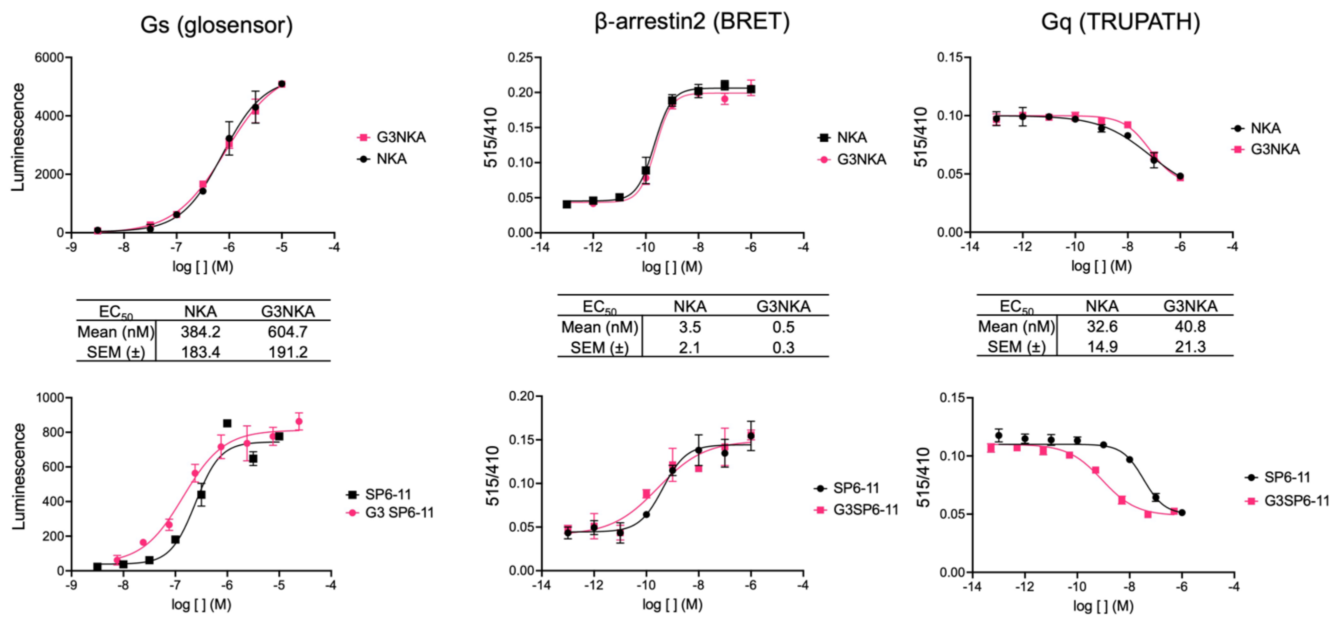

EC_{50}	NKA	G3NKA
Mean (nM)	384.2	604.7
SEM (±)	183.4	191.2

EC_{50}	NKA	G3NKA
Mean (nM)	3.5	0.5
SEM (±)	2.1	0.3

EC_{50}	NKA	G3NKA
Mean (nM)	32.6	40.8
SEM (±)	14.9	21.3

EC_{50}	SP6-11	G3SP6-11
Mean (nM)	231.1	102.5
SEM (±)	71.5	20.6

EC_{50}	SP6-11	G3SP6-11
Mean (nM)	0.4	0.2
SEM (±)	0.1	0.1

EC_{50}	SP6-11	G3SP6-11
Mean (nM)	27.5	0.3
SEM (±)	14.3	0.2

Figure S2. Comparison of the activity NKA and $SP_{6\text{-}11}$ to their triglycine analogues for inducing Gs, β-arrestin2 and G_q responses. Representative concentration-response curves (mean ± SD from technical replicates). EC_{50} was calculated from the fitting of a sigmoidal concentration-response model to data from three or more independent experiments using GraphPad Prism.

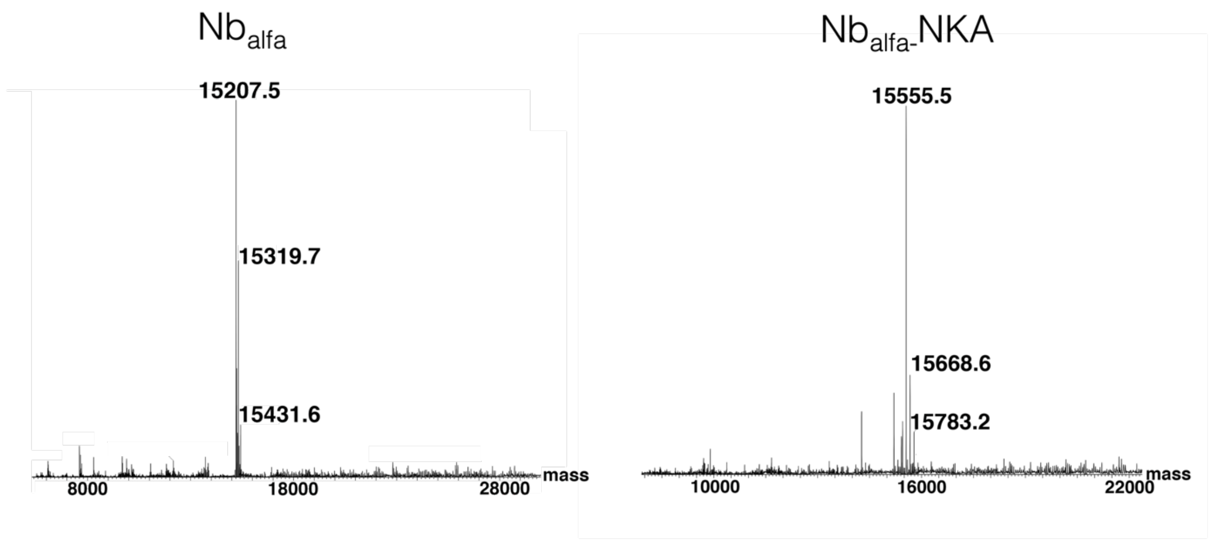

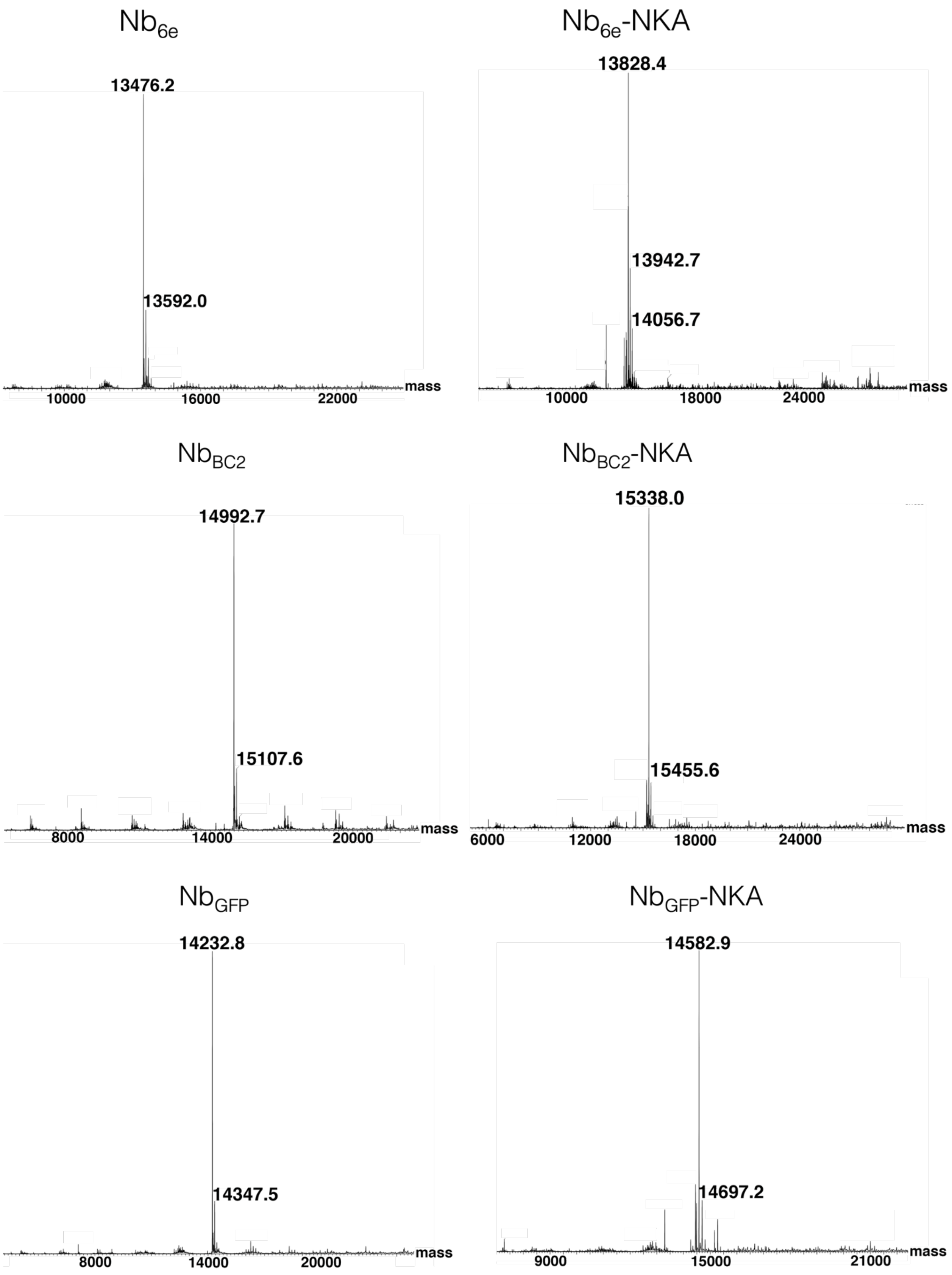

Nb_{6e}
13476.2
13592.0
mass
10000
16000
22000
Nb_{6e}-NKA
13828.4
13942.7
14056.7
mass
10000
18000
24000
Nb_{BC2}
14992.7
15107.6
mass
8000
14000
20000
Nb_{BC2}-NKA
15338.0
15455.6
mass
6000
12000
18000
24000
Nb_{GFP}
14232.8
14347.5
mass
8000
14000
20000
Nb_{GFP}-NKA
14582.9
14697.2
mass
9000
15000
21000

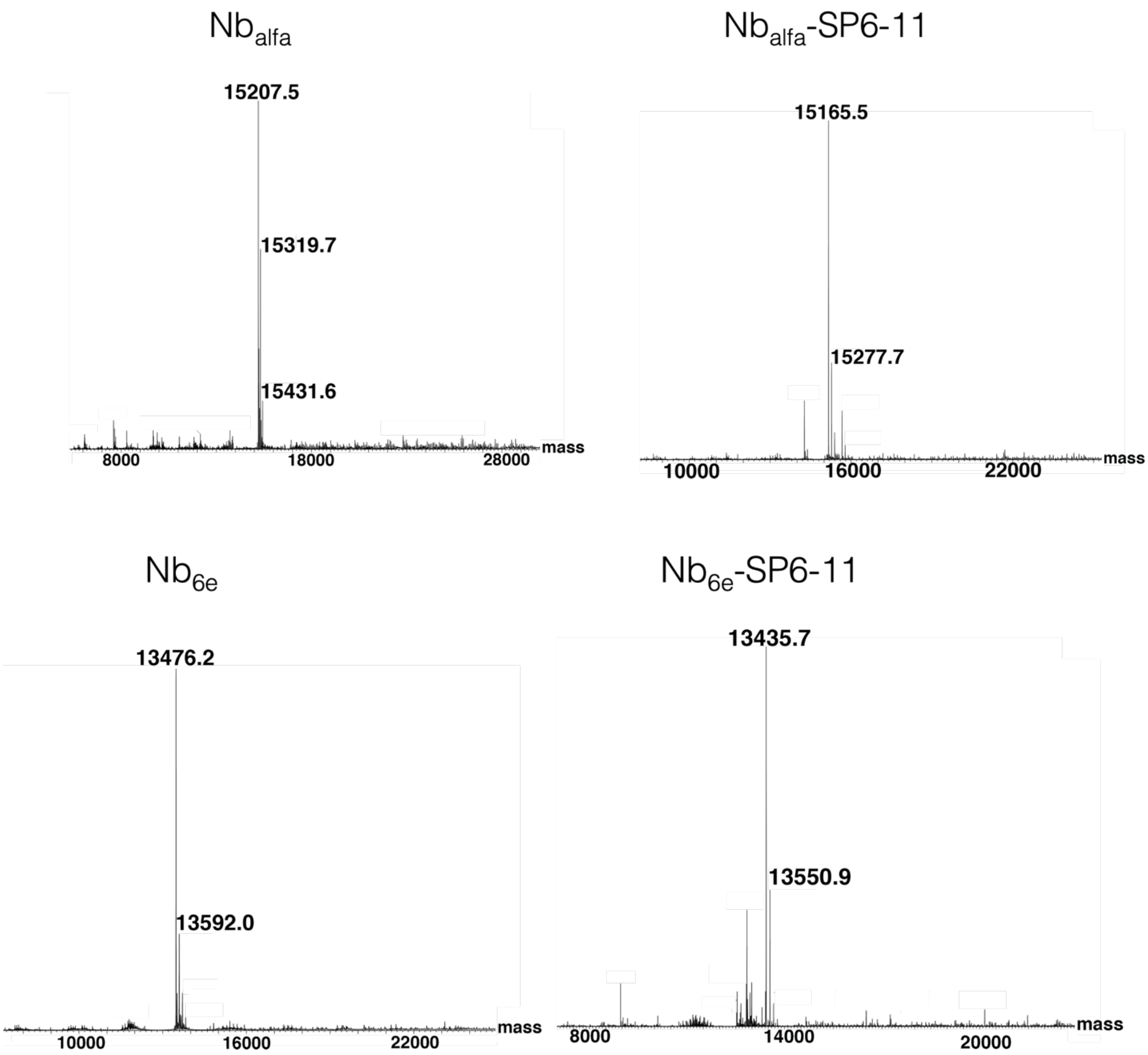

Nbalfa
15207.5
15319.7
15431.6
8000
18000
28000
mass
Nbalfa-SP6-11
15165.5
15277.7
10000
16000
22000
mass
Nb6e
13476.2
13592.0
10000
16000
22000
mass
Nb6e-SP6-11
13435.7
13550.9
8000
14000
20000
mass

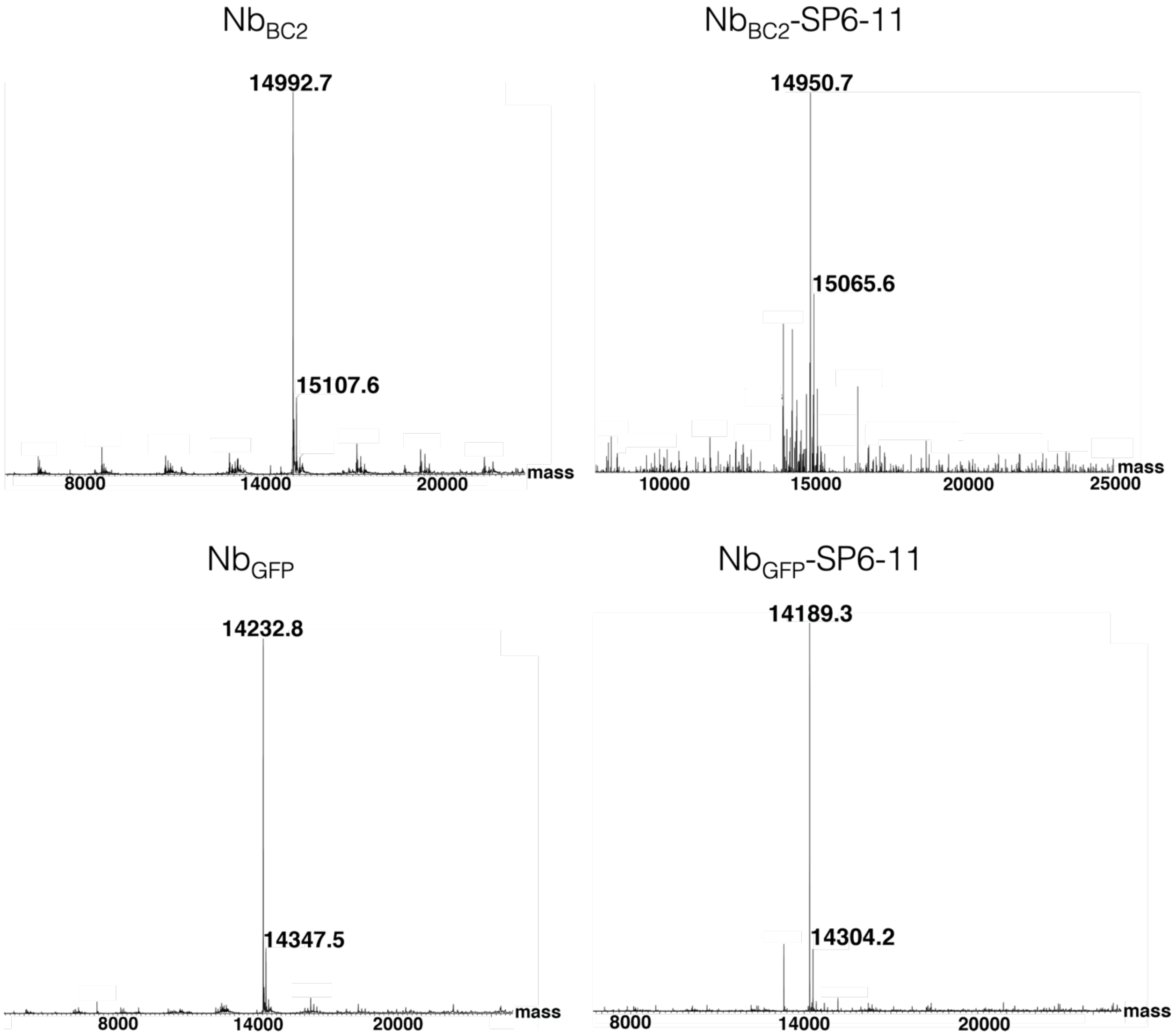

Figure S3. MS of the nanobodies before and after conjugation to G3NKA or G3SP$_{6-11}$. Mass spectra and deconvoluted masses were acquired according to Methods.

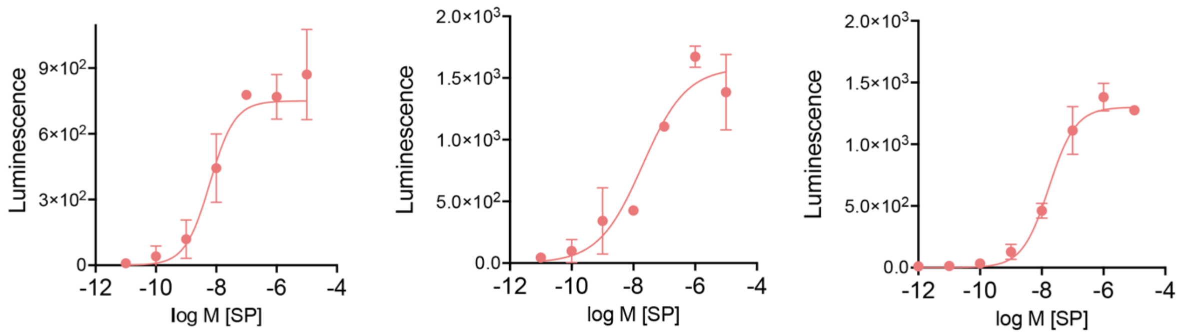

Figure S4. Replicate experiments of substance P (SP) activation of epitope-tagged NK1R via Glosensor assay. Data points correspond to mean ± SD from technical replicates.

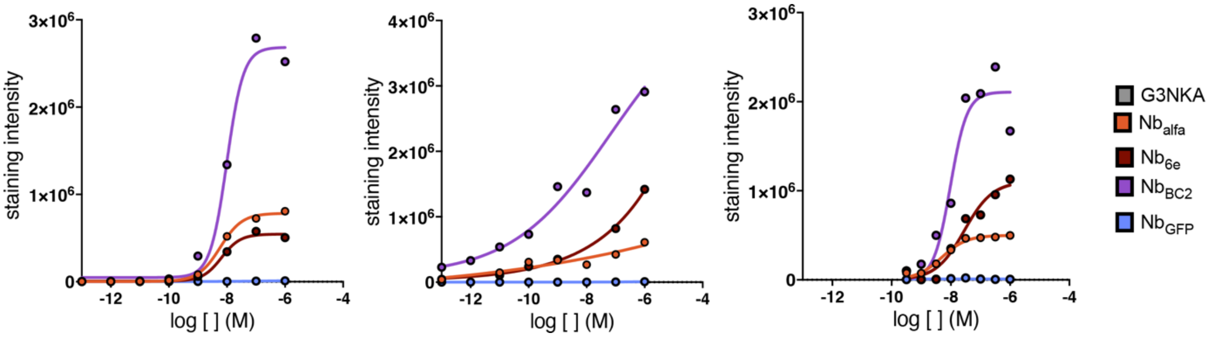

Figure S5. Independent replicates for flow cytometry analysis of biotin-labeled nanobodies binding to epitope-tagged NK1R cells. Staining and analysis was performed according to Methods.

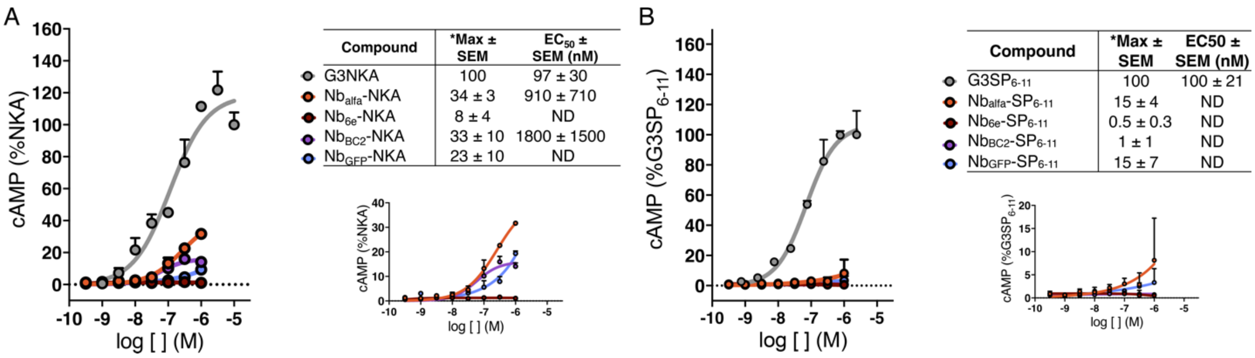

Compound	*Max ± SEM	EC$_{50}$ ± SEM (nM)
G3NKA	100	97 ± 30
Nb$_{alfa}$-NKA	34 ± 3	910 ± 710
Nb$_{6e}$-NKA	8 ± 4	ND
Nb$_{BC2}$-NKA	33 ± 10	1800 ± 1500
Nb$_{GFP}$-NKA	23 ± 10	ND

Compound	*Max ± SEM	EC50 ± SEM (nM)
G3SP$_{6-11}$	100	100 ± 21
Nb$_{alfa}$-SP$_{6-11}$	15 ± 4	ND
Nb$_{6e}$-SP$_{6-11}$	0.5 ± 0.3	ND
Nb$_{BC2}$-SP$_{6-11}$	1 ± 1	ND
Nb$_{GFP}$-SP$_{6-11}$	15 ± 7	ND

Figure S6. Effects of peptides and conjugates on cAMP production in NK1R wild-type cells. HEK293 cells stably transfected with Glosensor cAMP reporter (Promega Corp.)[23] and wild-type NK1R were treated with varying concentrations (1 pM – 10 mM) of the indicated peptides or conjugates. Activation was assessed by cAMP production after 6 min (n = 3) as described in Methods. (**A, B)** Representative concentration-response curves (mean ± SD). Insets of concentration-responses without G3-peptides included are shown (under table) to allow better visualization of the activity of the weaker compounds. Curves result from the fitting of a sigmoidal concentration-response model to data. *Max activity values were calculated by normalizing the response at 1 mM for conjugates to that of G3NKA or G3SP$_{6-11}$ at 10 mM (n = 3). EC$_{50}$ for conjugates with maximal responses lower than 30% were not determined (ND).

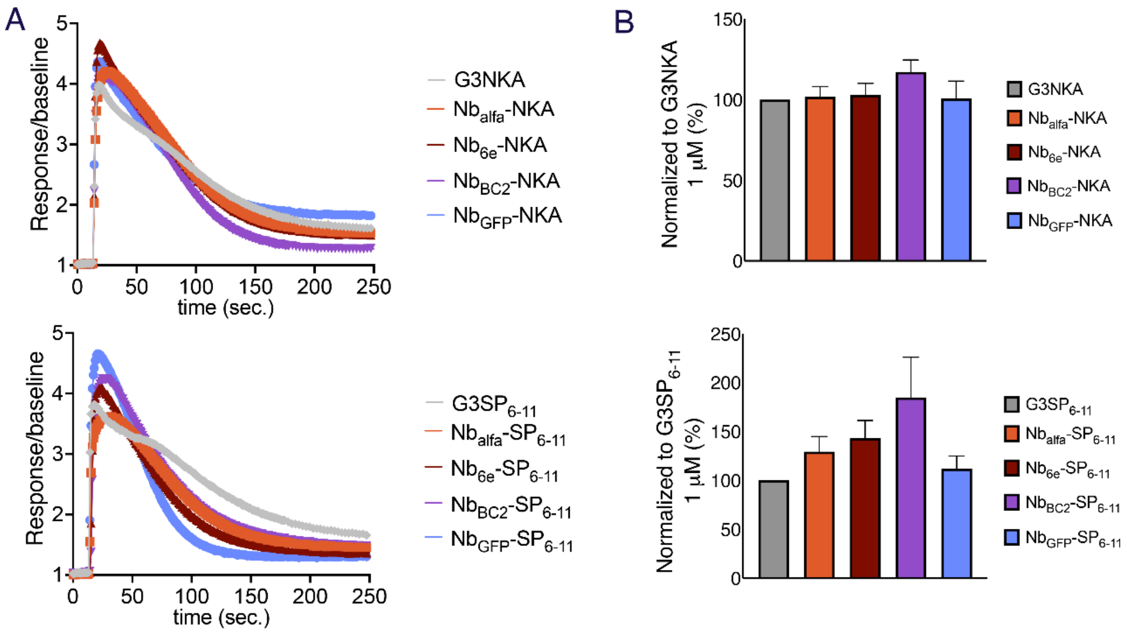

Figure S7. Effects of peptides and conjugates on intracellular Ca^{2+} mobilization. HEK293 cells stably expressing tagged NK1R were treated the indicated peptides or conjugates. **A.** Representative FLIPR traces of the fluorescenc over the basal fluorescence following the addition of the compounds at 1 μM to the cells. **B.** Bar graphs represent meai from three independent biological replicates. Data were normalized to G3NKA or $G3SP_{6-11}$ responses at 1 μM.

A

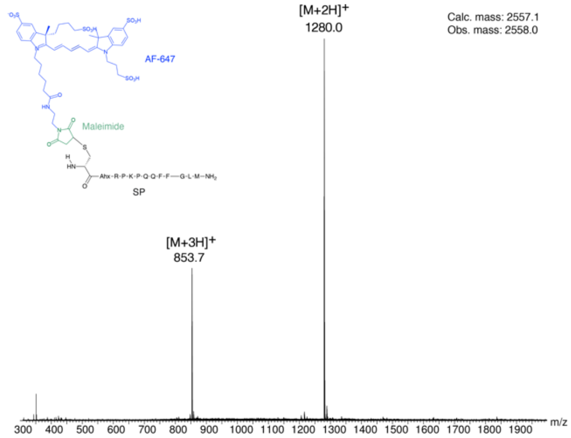

B

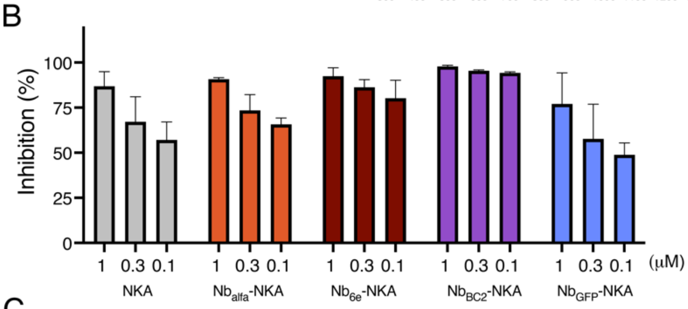

% inhibition ± SEM		1 uM	300 nM	100 nM
	NKA	87 ± 5	67 ± 8	57 ± 6
	Nb_{alfa}-NKA	91 ± 1	74 ± 6	66 ± 2
	Nb_{6e}-NKA	92 ± 3	86 ± 2 (*)	80 ± 6 (**)
	Nb_{BC2}-NKA	98 ± 0	95 ± 0 (***)	94 ± 0 (****)
	Nb_{GFP}-NKA	77 ± 10	58 ± 14	49 ± 4

( ) Dunnett's multiple comparison test vs NKA

C

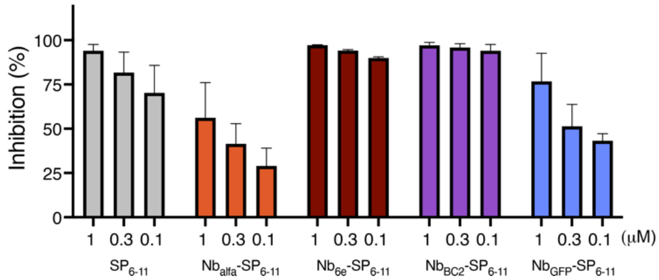

% inhibition ± SEM		1 uM	300 nM	100 nM
	SP_{6-11}	94 ± 2	82 ± 7	70 ± 9
	Nb_{alfa}-SP_{6-11}	56 ± 11 (****)	41 ± 7 (****)	29 ± 6 (****)
	Nb_{6e}-SP_{6-11}	97 ± 0	94 ± 0	90 ± 1
	Nb_{BC2}-SP_{6-11}	97 ± 1	96 ± 1	94 ± 2 (**)
	Nb_{GFP}-SP_{6-11}	77 ± 9	51 ± 7 (***)	43 ± 2 (**)

( ) Dunnett's multiple comparison test vs NKA

Figure S8. B**inding assay for measurement of Nb-ligand conjugate competition with labeled SP. (A)** Chemical structure and high-resolution MS analysis of SP-AF647. Binding assay was performed using flow cytometry to assess the ability of **(B)** NKA and Nb-NKA or **(C)** SP_{6-11} and Nb-SP_{6-11} to outcompete fluorescently labeled SP for binding to epitope-tagged NK1R. Values correspond to mean ± SEM from 3 biological replicates. Differences were evaluated for statistical significance with a one-way ANOVA followed by Dunnett correction. *p < 0.03, **p < 0.002, ***p < 0.0002, **** p < 0.0001

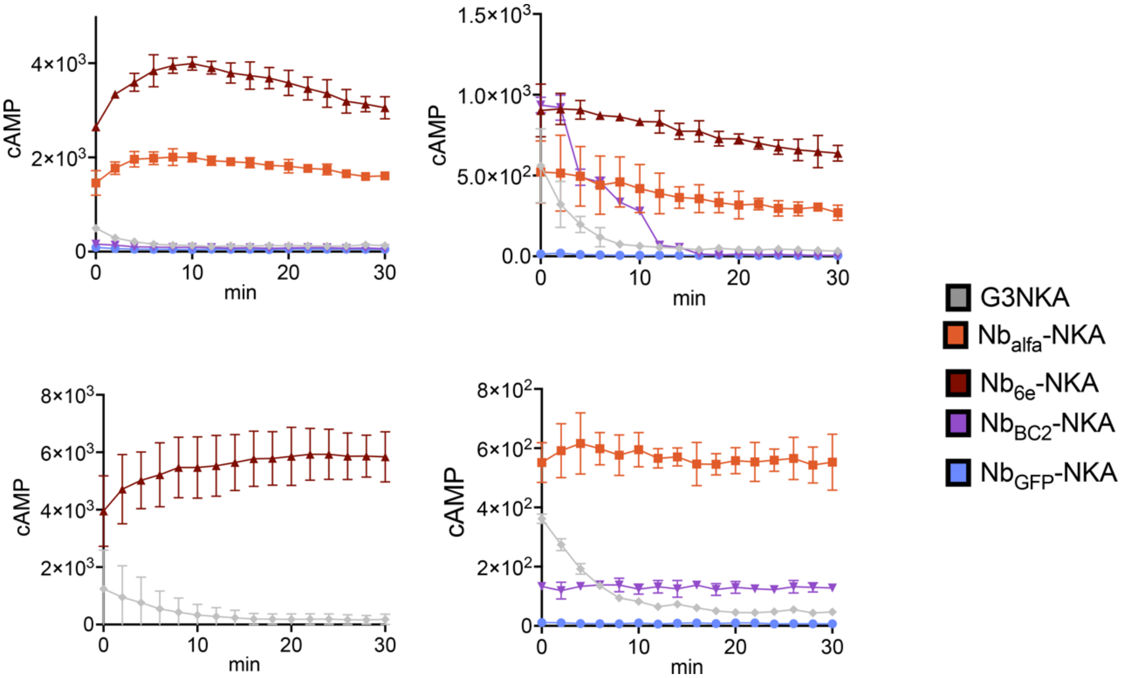

Figure S9. Replicate experiments for measurement of the decay of cAMP production via washout assay in epitope-tagged NK1R. Data points correspond to mean ± SD from technical replicates.

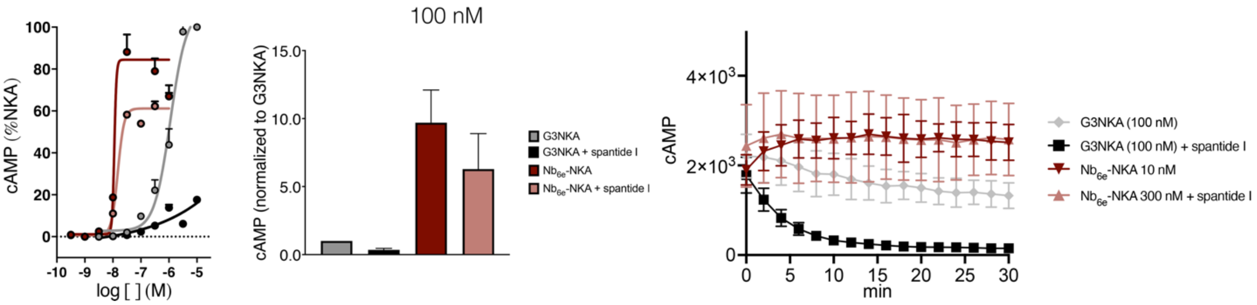

Figure S10. Impact of spantide I on the dissipation of cAMP signaling (washout) for Nb_{6e}-NKA and G3NKA. Excess/unbound ligand was removed, fresh media with 1 μM spantide I was added, and cAMP concentration was measured for an additional 30 min (washout) (n = 3). Washout assay data is summarized as the area under the curve (AUC) recorded for a 100 nM of peptide or conjugate. Data represent mean ± SEM from n=3 independent experiments. Representative concentration-response curves or cAMP production kinetics (mean ± SD) for washout assays.

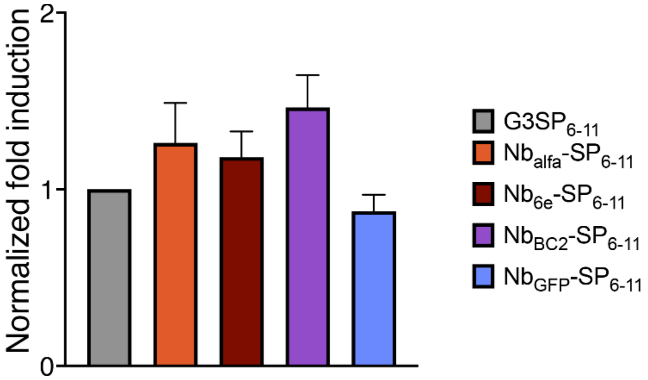

Figure S11. Evaluation of G3SP6-11 peptide and conjugate performance on Gq transcription. Transcriptional responses were evaluated through transfection of cells expressing epitope tagged NK1R with a luciferase reporter plasmid reporting on G_q signaling. Cells were incubated with ~35 nM of the indicated peptides or conjugates for 17 hours and transcription was measured as described in Methods. Data represent mean ± SEM from n=3 independent experiments.

Plasmids and nanobody sequences

Epitope-tagged NK1R (receptor sequence underlined)

gacggatcgggagatctcccgatcccctatggtgcactctcagtacaatctgctctgatgccgcatagttaagccagtatctgctccctgcttgtgtgt
tggaggtcgctgagtagtgcgcgagcaaaatttaagctacaacaaggcaaggcttgaccgacaattgcatgaagaatctgcttagggttaggcg
ttttgcgctgcttcgcgatgtacgggccagatatacgcgttgacattgattattgactagttattaatagtaatcaattacggggtcattagttcatagcc
catatatggagttccgcgttacataacttacggtaaatggcccgcctggctgaccgcccaacgacccccgcccattgacgtcaataatgacgtat
gttcccatagtaacgccaatagggactttccattgacgtcaatgggtggagtatttacggtaaactgcccacttggcagtacatcaagtgtatcatat
gccaagtacgccccctattgacgtcaatgacggtaaatggcccgcctggcattatgcccagtacatgaccttatgggactttcctacttggcagtac
atctacgtattagtcatcgctattaccatggtgatgcggttttggcagtacatcaatgggcgtggatagcggtttgactcacggggatttccaagtctcc
accccattgacgtcaatgggagtttgttttggcaccaaaatcaacgggactttccaaaatgtcgtaacaactccgccccattgacgcaaatgggcg
gtaggcgtgtacggtgggaggtctatataagcagagctctctggctaactagagaacccactgcttactggcttatcgaaattaatacgactcacta
tagggagacccaagctggctagcgtttaaacttaagcttggtaccgagctcggatccgccaccatgaagacaatcatcgccctgagctacatctt
ctgcctggtgttcgccggaccttctagactggaagaggagctgcgccggagactgaccgagcccggccaggccgaccaggaggccaagga
actggctagacagatcagcggccctgatagagtgcgggccgtgtcccactggagcagcatggataacgtcctcccggtggactcagacctctc
cccaaacatctccactaacacctcggaacccaatcagttcgtgcaaccagcctggcaaattgtcctttgggcagctgcctacacggtcattgtggt
gacctctgtggtgggcaacgtggtagtgatgtggatcatcttagcccacaaaagaatgaggacagtgacgaactattttctggtgaacctggcctt
cgcggaggcctccatggctgcattcaatacagtggtgaacttcacctatgctgtccacaacgaatggtactacggcctgttctactgcaagttccac
aacttctttcccatcgccgctgtcttcgccagtatctactccatgacggctgtggcctttgataggtacatggccatcatacatcccctccagccccgg
ctgtcagccacagccaccaaagtggtcatctgtgtcatctgggtcctggctctcctgctggccttcccccagggctactactcaaccacagagacc
atgcccagcagagtcgtgtgcatgatcgaatggccagagcatccgaacaagatttatgagaaagtgtaccacatctgtgtgactgtgctgatctac
ttcctcccccctgctggtgattggctatgcatacaccgtagtgggaatcacactatgggccagtgagatccccggggactcctctgaccgctaccac
gagcaagtctctgccaagcgcaaggtggtcaaaatgatgattgtcgtggtgtgcaccttcgccatctgctggctgcccttccacatcttcttcctcctg
ccctacatcaacccagatctctacctgaagaagtttatccagcaggtctacctggccatcatgtggctggccatgagctccaccatgtacaacccc
atcatctactgctgcctcaatgacaggttccgtctgggcttcaagcatgccttccggtgctgccccttcatcagcgccggcgactatgaggggctgg
aaatgaaatccacccggtatctccagacccagggcagtgtgtacaaagtcagccgcctggagaccaccatctccacagtggtgggggcccac
gaggaggagccagaggacggccccaaggccacaccctcgtccctggacctgacctccaactgctcttcacgaagtgactccaagaccatga

cagagagcttcagcttctcctccaatgtgctctccgattacaaggatgacgacgataagtgataaacccgctgatcagcctcgactgtgccttctag
ttgccagccatctgttgtttgcccctcccccgtgccttccttgaccctggaaggtgccactcccactgtcctttcctaataaaatgaggaaattgcatcg
cattgtctgagtaggtgtcattctattctggggggtggggtggggcaggacagcaagggggaggattgggaagacaatagcaggcatgctggg
gatgcggtgggctctatggcttctgaggcggaaagaaccagctggggctctagggggtatccccacgcgccctgtagcggcgcattaagcgcg
gcgggtgtggtggttacgcgcagcgtgaccgctacacttgccagcgccctagcgcccgctcctttcgctttcttcccttcctttctcgccacgttcgcc
ggctttccccgtcaagctctaaatcgggggctccctttagggttccgatttagtgctttacggcacctcgaccccaaaaaacttgattagggtgatggt
tcacgtagtgggccatcgccctgatagacggtttttcgccctttgacgttggagtccacgttctttaatagtggactcttgttccaaactggaacaacac
tcaaccctatctcggtctattcttttgatttataagggattttgccgatttcggcctattggttaaaaaatgagctgatttaacaaaaatttaacgcgaatta
attctgtggaatgtgtgtcagttagggtgtggaaagtccccaggctccccagcaggcagaagtatgcaaagcatgcatctcaattagtcagcaac
caggtgtggaaagtccccaggctccccagcaggcagaagtatgcaaagcatgcatctcaattagtcagcaaccatagtcccgcccctaactcc
gcccatcccgcccctaactccgcccagttccgcccattctccgccccatggctgactaattttttttatttatgcagaggccgaggccgcctctgcctct
gagctattccagaagtagtgaggaggcttttttggaggcctaggcttttgcaaaaagctcccgggagcttgtatatccattttcggatctgatcaaga
gacaggatgaggatcgtttcgcatgattgaacaagatggattgcacgcaggttctccggccgcttgggtggagaggctattcggctatgactggg
cacaacagacaatcggctgctctgatgccgccgtgttccggctgtcagcgcaggggcgcccggttctttttgtcaagaccgacctgtccggtgccc
tgaatgaactgcaggacgaggcagcgcggctatcgtggctggccacgacgggcgttccttgcgcagctgtgctcgacgttgtcactgaagcgg
gaagggactggctgctattgggcgaagtgccggggcaggatctcctgtcatctcaccttgctcctgccgagaaagtatccatcatggctgatgca
atgcggcggctgcatacgcttgatccggctacctgcccattcgaccaccaagcgaaacatcgcatcgagcgagcacgtactcggatggaagcc
ggtcttgtcgatcaggatgatctggacgaagagcatcaggggctcgcgccagccgaactgttcgccaggctcaaggcgcgcatgcccgacgg
cgaggatctcgtcgtgacccatggcgatgcctgcttgccgaatatcatggtggaaaatggccgcttttctggattcatcgactgtggccggctgggt
gtggcggaccgctatcaggacatagcgttggctacccgtgatattgctgaagagcttggcggcgaatgggctgaccgcttcctcgtgctttacggt
atcgccgctcccgattcgcagcgcatcgccttctatcgccttcttgacgagttcttctgagcgggactctggggttcgaaatgaccgaccaagcgac
gcccaacctgccatcacgagatttcgattccaccgccgccttctatgaaaggttgggcttcggaatcgttttccgggacgccggctggatgatcctc
cagcgcggggatctcatgctggagttcttcgcccaccccaacttgtttattgcagcttataatggttacaaataaagcaatagcatcacaaatttcac
aaataaagcatttttttcactgcattctagttgtggtttgtccaaactcatcaatgtatcttatcatgtctgtataccgtcgacctctagctagagcttggcgt
aatcatggtcatagctgtttcctgtgtgaaattgttatccgctcacaattccacacaacatacgagccggaagcataaagtgtaaagcctggggtgc
ctaatgagtgagctaactcacattaattgcgttgcgctcactgcccgctttccagtcgggaaacctgtcgtgccagctgcattaatgaatcggccaa
cgcgcggggagaggcggtttgcgtattgggcgctcttccgcttcctcgctcactgactcgctgcgctcggtcgttcggctgcggcgagcggtatca
gctcactcaaaggcggtaatacggttatccacagaatcaggggataacgcaggaaagaacatgtgagcaaaaggccagcaaaaggccag
gaaccgtaaaaaggccgcgttgctggcgtttttccataggctccgcccccctgacgagcatcacaaaaatcgacgctcaagtcagaggtggcg
aaacccgacaggactataaagataccaggcgtttccccctggaagctccctcgtgcgctctcctgttccgaccctgccgcttaccggatacctgtc
cgcctttctcccttcgggaagcgtggcgctttctcatagctcacgctgtaggtatctcagttcggtgtaggtcgttcgctccaagctgggctgtgtgcac
gaaccccccgttcagcccgaccgctgcgccttatccggtaactatcgtcttgagtccaacccggtaagacacgacttatcgccactggcagcagc
cactggtaacaggattagcagagcgaggtatgtaggcggtgctacagagttcttgaagtggtggcctaactacggctacactagaagaacagta
tttggtatctgcgctctgctgaagccagttaccttcggaaaaagagttggtagctcttgatccggcaaacaaaccaccgctggtagcggtggtttttt
gtttgcaagcagcagattacgcgcagaaaaaaaggatctcaagaagatcctttgatcttttctacggggtctgacgctcagtggaacgaaaactc
acgttaagggattttggtcatgagattatcaaaaaggatcttcacctagatccttttaaattaaaaatgaagttttaaatcaatctaaagtatatatgagt
aaacttggtctgacagttaccaatgcttaatcagtgaggcacctatctcagcgatctgtctatttcgttcatccatagttgcctgactccccgtcgtgtag
ataactacgatacgggagggcttaccatctggccccagtgctgcaatgataccgcgagacccacgctcaccggctccagatttatcagcaataa
accagccagccggaagggccgagcgcagaagtggtcctgcaactttatccgcctccatccagtctattaattgttgccgggaagctagagtaagt
agttcgccagttaatagtttgcgcaacgttgttgccattgctacaggcatcgtggtgtcacgctcgtcgtttggtatggcttcattcagctccggttccca
acgatcaaggcgagttacatgatcccccatgttgtgcaaaaaagcggttagctccttcggtcctccgatcgttgtcagaagtaagttggccgcagt
gttatcactcatggttatggcagcactgcataattctcttactgtcatgccatccgtaagatgcttttctgtgactggtgagtactcaaccaagtcattctg
agaatagtgtatgcggcgaccgagttgctcttgcccggcgtcaatacgggataataccgcgccacatagcagaactttaaaagtgctcatcattg
gaaaacgttcttcggggcgaaaactctcaaggatcttaccgctgttgagatccagttcgatgtaacccactcgtgcacccaactgatcttcagcatc
ttttactttcaccagcgtttctgggtgagcaaaaacaggaaggcaaaatgccgcaaaaaagggaataagggcgacacggaaatgttgaatact
catactcttcctttttcaatattattgaagcatttatcagggttattgtctcatgagcggatacatatttgaatgtatttagaaaaataaacaaataggggtt
ccgcgcacatttccccgaaaagtgccacctgacgtc

Wild-type NK1R (receptor sequence underlined)

gacggatcgggagatctcccgatcccctatggtgcactctcagtacaatctgctctgatgccgcatagttaagccagtatctgctccctgcttgtgtgt
tggaggtcgctgagtagtgcgcgagcaaaatttaagctacaacaaggcaaggcttgaccgacaattgcatgaagaatctgcttagggttaggcg
ttttgcgctgcttcgcgatgtacgggccagatatacgcgttgacattgattattgactagttattaatagtaatcaattacggggtcattagttcatagcc
catatatggagttccgcgttacataacttacggtaaatggcccgcctggctgaccgcccaacgacccccgcccattgacgtcaataatgacgtat
gttcccatagtaacgccaatagggactttccattgacgtcaatgggtggagtatttacggtaaactgcccacttggcagtacatcaagtgtatcatat
gccaagtacgccccctattgacgtcaatgacggtaaatggcccgcctggcattatgcccagtacatgaccttatgggactttcctacttggcagtac
atctacgtattagtcatcgctattaccatggtgatgcggttttggcagtacatcaatgggcgtggatagcggtttgactcacggggatttccaagtctcc
accccattgacgtcaatgggagtttgttttggcaccaaaatcaacgggactttccaaaatgtcgtaacaactccgccccattgacgcaaatgggcg
gtaggcgtgtacggtgggaggtctatataagcagagctctctggctaactagagaacccactgcttactggcttatcgaaattaatacgactcacta
tagggagacccaagctggctagcgtttaaacttaagcttggtaccgagctcggatccgccaccatggataacgtcctcccggtggactcagacct
ctccccaaacatctccactaacacctcggaacccaatcagttcgtgcaaccagcctggcaaattgtcctttgggcagctgcctacacggtcattgt
ggtgacctctgtggtgggcaacgtggtagtgatgtggatcatcttagcccacaaaagaatgaggacagtgacgaactattttctggtgaacctggc
cttcgcggaggcctccatggctgcattcaatacagtggtgaacttcacctatgctgtccacaacgaatggtactacggcctgttctactgcaagttcc
acaacttctttcccatcgccgctgtcttcgccagtatctactccatgacggctgtggcctttgataggtacatggccatcatacatcccctccagcccc
ggctgtcagccacagccaccaaagtggtcatctgtgtcatctgggtcctggctctcctgctggccttcccccagggctactactcaaccacagaga
ccatgcccagcagagtcgtgtgcatgatcgaatggccagagcatccgaacaagatttatgagaaagtgtaccacatctgtgtgactgtgctgatct
acttcctcccccctgctggtgattggctatgcatacaccgtagtgggaatcacactatgggccagtgagatccccggggactcctctgaccgctacc
acgagcaagtctctgccaagcgcaaggtggtcaaaatgatgattgtcgtggtgtgcaccttcgccatctgctggctgcccttccacatcttcttcctcc
tgccctacatcaacccagatctctacctgaagaagtttatccagcaggtctacctggccatcatgtggctggccatgagctccaccatgtacaacc
ccatcatctactgctgcctcaatgacaggttccgtctgggcttcaagcatgccttccggtgctgccccttcatcagcgccggcgactatgaggggct
ggaaatgaaatccacccggtatctccagacccagggcagtgtgtacaaagtcagccgcctggagaccaccatctccacagtggtgggggccc
acgaggaggagccagaggacggccccaaggccacaccctcgtccctggacctgacctccaactgctcttcacgaagtgactccaagaccat
gacagagagcttcagcttctcctccaatgtgctctccgattacaaggatgacgacgataagtgataaacccgctgatcagcctcgactgtgccttct
agttgccagccatctgttgtttgcccctcccccgtgccttccttgaccctggaaggtgccactcccactgtcctttcctaataaaatgaggaaattgcat
cgcattgtctgagtaggtgtcattctattctggggggtggggtggggcaggacagcaagggggaggattgggaagacaatagcaggcatgctg
gggatgcggtgggctctatggcttctgaggcggaaagaaccagctggggctctagggggtatccccacgcgccctgtagcggcgcattaagcg
cggcgggtgtggtggttacgcgcagcgtgaccgctacacttgccagcgccctagcgcccgctcctttcgctttcttcccttcctttctcgccacgttcg
ccggctttccccgtcaagctctaaatcgggggctccctttagggttccgatttagtgctttacggcacctcgaccccaaaaaacttgattagggtgat
ggttcacgtagtgggccatcgccctgatagacggtttttcgccctttgacgttggagtccacgttctttaatagtggactcttgttccaaactggaacaa
cactcaaccctatctcggtctattcttttgatttataagggattttgccgatttcggcctattggttaaaaaatgagctgatttaacaaaaatttaacgcga
attaattctgtggaatgtgtgtcagttagggtgtggaaagtccccaggctccccagcaggcagaagtatgcaaagcatgcatctcaattagtcagc
aaccaggtgtggaaagtccccaggctccccagcaggcagaagtatgcaaagcatgcatctcaattagtcagcaaccatagtcccgcccctaa
ctccgcccatcccgcccctaactccgcccagttccgcccattctccgccccatggctgactaattttttttatttatgcagaggccgaggccgcctctg
cctctgagctattccagaagtagtgaggaggcttttttggaggcctaggcttttgcaaaaagctcccgggagcttgtatatccattttcggatctgatca
agagacaggatgaggatcgtttcgcatgattgaacaagatggattgcacgcaggttctccggccgcttgggtggagaggctattcggctatgact
gggcacaacagacaatcggctgctctgatgccgccgtgttccggctgtcagcgcaggggcgcccggttctttttgtcaagaccgacctgtccggt
gccctgaatgaactgcaggacgaggcagcgcggctatcgtggctggccacgacgggcgttccttgcgcagctgtgctcgacgttgtcactgaag
cgggaagggactggctgctattgggcgaagtgccggggcaggatctcctgtcatctcaccttgctcctgccgagaaagtatccatcatggctgat
gcaatgcggcggctgcatacgcttgatccggctacctgcccattcgaccaccaagcgaaacatcgcatcgagcgagcacgtactcggatgga
agccggtcttgtcgatcaggatgatctggacgaagagcatcaggggctcgcgccagccgaactgttcgccaggctcaaggcgcgcatgcccg
acggcgaggatctcgtcgtgacccatggcgatgcctgcttgccgaatatcatggtggaaaatggccgcttttctggattcatcgactgtggccggct
gggtgtggcggaccgctatcaggacatagcgttggctacccgtgatattgctgaagagcttggcggcgaatgggctgaccgcttcctcgtgcttta
cggtatcgccgctcccgattcgcagcgcatcgccttctatcgccttcttgacgagttcttctgagcgggactctggggttcgaaatgaccgaccaag
cgacgcccaacctgccatcacgagatttcgattccaccgccgccttctatgaaaggttgggcttcggaatcgttttccgggacgccggctggatga
tcctccagcgcggggatctcatgctggagttcttcgcccaccccaacttgtttattgcagcttataatggttacaaataaagcaatagcatcacaaatt
tcacaaataaagcatttttttcactgcattctagttgtggtttgtccaaactcatcaatgtatcttatcatgtctgtataccgtcgacctctagctagagcttg
gcgtaatcatggtcatagctgtttcctgtgtgaaattgttatccgctcacaattccacacaacatacgagccggaagcataaagtgtaaagcctggg
gtgcctaatgagtgagctaactcacattaattgcgttgcgctcactgcccgctttccagtcgggaaacctgtcgtgccagctgcattaatgaatcggc
caacgcgcggggagaggcggtttgcgtattgggcgctcttccgcttcctcgctcactgactcgctgcgctcggtcgttcggctgcggcgagcggta
tcagctcactcaaaggcggtaatacggttatccacagaatcaggggataacgcaggaaagaacatgtgagcaaaaggccagcaaaaggcc
aggaaccgtaaaaaggccgcgttgctggcgtttttccataggctccgcccccctgacgagcatcacaaaaatcgacgctcaagtcagaggtgg

cgaaacccgacaggactataaagataccaggcgtttccccctggaagctccctcgtgcgctctcctgttccgaccctgccgcttaccggatacctg
tccgcctttctcccttcgggaagcgtggcgctttctcatagctcacgctgtaggtatctcagttcggtgtaggtcgttcgctccaagctgggctgtgtgc
acgaaccccccgttcagcccgaccgctgcgccttatccggtaactatcgtcttgagtccaacccggtaagacacgacttatcgccactggcagca
gccactggtaacaggattagcagagcgaggtatgtaggcggtgctacagagttcttgaagtggtggcctaactacggctacactagaagaaca
gtatttggtatctgcgctctgctgaagccagttaccttcggaaaaagagttggtagctcttgatccggcaaacaaaccaccgctggtagcggtggttt
ttttgtttgcaagcagcagattacgcgcagaaaaaaaggatctcaagaagatcctttgatcttttctacggggtctgacgctcagtggaacgaaaa
ctcacgttaagggattttggtcatgagattatcaaaaaggatcttcacctagatccttttaaattaaaaatgaagttttaaatcaatctaaagtatatatg
agtaaacttggtctgacagttaccaatgcttaatcagtgaggcacctatctcagcgatctgtctatttcgttcatccatagttgcctgactccccgtcgtg
tagataactacgatacgggagggcttaccatctggccccagtgctgcaatgataccgcgagacccacgctcaccggctccagatttatcagcaa
taaaccagccagccggaagggccgagcgcagaagtggtcctgcaactttatccgcctccatccagtctattaattgttgccgggaagctagagta
agtagttcgccagttaatagtttgcgcaacgttgttgccattgctacaggcatcgtggtgtcacgctcgtcgtttggtatggcttcattcagctccggttc
ccaacgatcaaggcgagttacatgatcccccatgttgtgcaaaaaagcggttagctccttcggtcctccgatcgttgtcagaagtaagttggccgc
agtgttatcactcatggttatggcagcactgcataattctcttactgtcatgccatccgtaagatgcttttctgtgactggtgagtactcaaccaagtcatt
ctgagaatagtgtatgcggcgaccgagttgctcttgcccggcgtcaatacgggataataccgcgccacatagcagaactttaaaagtgctcatca
ttggaaaacgttcttcggggcgaaaactctcaaggatcttaccgctgttgagatccagttcgatgtaacccactcgtgcacccaactgatcttcagc
atcttttactttcaccagcgtttctgggtgagcaaaaacaggaaggcaaaatgccgcaaaaaagggaataagggcgacacggaaatgttgaat
actcatactcttcctttttcaatattattgaagcatttatcagggttattgtctcatgagcggatacatatttgaatgtatttagaaaaataaacaaatagg
ggttccgcgcacatttccccgaaaagtgccacctgacgtc

Nb$_{6e}$
tggcgaatgggacgcgccctgtagcggcgcattaagcgcggcgggtgtggtggttacgcgcagcgtgaccgctacacttgccagcg
ccctagcgcccgctcctttcgctttcttcccttcctttctcgccacgttcgccggctttccccgtcaagctctaaatcgggggctccctttagg
gttccgatttagtgctttacggcacctcgaccccaaaaaacttgattagggtgatggttcacgtagtgggccatcgccctgatagacggtt
tttcgccctttgacgttggagtccacgttctttaatagtggactcttgttccaaactggaacaacactcaaccctatctcggtctattcttttgatt
tataagggattttgccgatttcggcctattggttaaaaaatgagctgatttaacaaaaatttaacgcgaattttaacaaaatattaacgttta
caatttcaggtggcacttttcggggaaatgtgcgcggaacccctatttgtttatttttctaaatacattcaaatatgtatccgctcatgaattaa
ttcttagaaaaactcatcgagcatcaaatgaaactgcaatttattcatatcaggattatcaataccatatttttgaaaaagccgtttctgtaat
gaaggagaaaactcaccgaggcagttccataggatggcaagatcctggtatcggtctgcgattccgactcgtccaacatcaatacaa
cctattaatttcccctcgtcaaaaataaggttatcaagtgagaaatcaccatgagtgacgactgaatccggtgagaatggcaaaagttt
atgcatttctttccagacttgttcaacaggccagccattacgctcgtcatcaaaatcactcgcatcaaccaaaccgttattcattcgtgattg
cgcctgagcgagacgaaatacgcgatcgctgttaaaaggacaattacaaacaggaatcgaatgcaaccggcgcaggaacactgc
cagcgcatcaacaatattttcacctgaatcaggatattcttctaatacctggaatgctgttttcccggggatcgcagtggtgagtaaccatg
catcatcaggagtacggataaaatgcttgatggtcggaagaggcataaattccgtcagccagtttagtctgaccatctcatctgtaacat
cattggcaacgctacctttgccatgtttcagaaacaactctggcgcatcgggcttcccatacaatcgatagattgtcgcacctgattgccc
gacattatcgcgagcccatttatacccatataaatcagcatccatgttggaatttaatcgcggcctagagcaagacgtttcccgttgaata
tggctcataacaccccttgtattactgtttatgtaagcagacagttttattgttcatgaccaaaatcccttaacgtgagttttcgttccactgag
cgtcagaccccgtagaaaagatcaaaggatcttcttgagatcctttttttctgcgcgtaatctgctgcttgcaaacaaaaaaaccaccgc
taccagcggtggtttgtttgccggatcaagagctaccaactctttttccgaaggtaactggcttcagcagagcgcagataccaaatactg
tccttctagtgtagccgtagttaggccaccacttcaagaactctgtagcaccgcctacatacctcgctctgctaatcctgttaccagtggct
gctgccagtggcgataagtcgtgtcttaccgggttggactcaagacgatagttaccggataaggcgcagcggtcgggctgaacggg
gggttcgtgcacacagcccagcttggagcgaacgacctacaccgaactgagatacctacagcgtgagctatgagaaagcgccacg
cttcccgaagggagaaaggcggacaggtatccggtaagcggcagggtcggaacaggagagcgcacgagggagcttccagggg
gaaacgcctggtatctttatagtcctgtcgggtttcgccacctctgacttgagcgtcgatttttgtgatgctcgtcaggggggcggagcctat
ggaaaaacgccagcaacgcggcctttttacggttcctggccttttgctggccttttgctcacatgttctttcctgcgttatcccctgattctgtg
gataaccgtattaccgcctttgagtgagctgataccgctcgccgcagccgaacgaccgagcgcagcgagtcagtgagcgaggaag
cggaagagcgcctgatgcggtattttctccttacgcatctgtgcggtatttcacaccgcatatatggtgcactctcagtacaatctgctctga

tgccgcatagttaagccagtatacactccgctatcgctacgtgactgggtcatggctgcgccccgacacccgccaacacccgctgac
gcgccctgacgggcttgtctgctcccggcatccgcttacagacaagctgtgaccgtctccgggagctgcatgtgtcagaggttttcacc
gtcatcaccgaaacgcgcgaggcagctgcggtaaagctcatcagcgtggtcgtgaagcgattcacagatgtctgcctgttcatccgcg
tccagctcgttgagtttctccagaagcgttaatgtctggcttctgataaagcgggccatgttaagggcggtttttcctgtttggtcactgatgc
ctccgtgtaagggggatttctgttcatgggggtaatgataccgatgaaacgagagaggatgctcacgatacgggttactgatgatgaa
catgcccggttactggaacgttgtgagggtaaacaactggcggtatggatgcggcgggaccagagaaaaatcactcagggtcaatg
ccagcgcttcgttaatacagatgtaggtgttccacagggtagccagcagcatcctgcgatgcagatccggaacataatggtgcaggg
cgctgacttccgcgtttccagactttacgaaacacggaaaccgaagaccattcatgttgttgctcaggtcgcagacgttttgcagcagca
gtcgcttcacgttcgctcgcgtatcggtgattcattctgctaaccagtaaggcaaccccgccagcctagccgggtcctcaacgacagga
gcacgatcatgcgcacccgtggggccgccatgccggcgataatggcctgcttctcgccgaaacgtttggtggcgggaccagtgacg
aaggcttgagcgagggcgtgcaagattccgaataccgcaagcgacaggccgatcatcgtcgcgctccagcgaaagcggtcctcgc
cgaaaatgacccagagcgctgccggcacctgtcctacgagttgcatgataaagaagacagtcataagtgcggcgacgatagtcatg
ccccgcgcccaccggaaggagctgactgggttgaaggctctcaagggcatcggtcgagatcccggtgcctaatgagtgagctaactt
acattaattgcgttgcgctcactgcccgctttccagtcgggaaacctgtcgtgccagctgcattaatgaatcggccaacgcgcggggag
aggcggtttgcgtattgggcgccagggtggtttttcttttcaccagtgagacgggcaacagctgattgcccttcaccgcctggccctgag
agagttgcagcaagcggtccacgctggtttgccccagcaggcgaaaatcctgtttgatggtggttaacggcgggatataacatgagct
gtcttcggtatcgtcgtatcccactaccgagatatccgcaccaacgcgcagcccggactcggtaatggcgcgcattgcgcccagcgc
catctgatcgttggcaaccagcatcgcagtgggaacgatgccctcattcagcatttgcatggtttgttgaaaaccggacatggcactcc
agtcgccttcccgttccgctatcggctgaatttgattgcgagtgagatatttatgccagccagccagacgcagacgcgccgagacaga
acttaatgggcccgctaacagcgcgatttgctggtgacccaatgcgaccagatgctccacgcccagtcgcgtaccgtcttcatgggag
aaaataatactgttgatgggtgtctggtcagagacatcaagaaataacgccggaacattagtgcaggcagcttccacagcaatggca
tcctggtcatccagcggatagttaatgatcagcccactgacgcgttgcgcgagaagattgtgcaccgccgctttacaggcttcgacgcc
gcttcgttctaccatcgacaccaccacgctggcacccagttgatcggcgcgagatttaatcgccgcgacaatttgcgacggcgcgtgc
agggccagactggaggtggcaacgccaatcagcaacgactgtttgcccgccagttgttgtgccacgcggttgggaatgtaattcagct
ccgccatcgccgcttccactttttcccgcgttttcgcagaaacgtggctggcctggttcaccacgcgggaaacggtctgataagagaca
ccggcatactctgcgacatcgtataacgttactggtttcacattcaccaccctgaattgactctcttccgggcgctatcatgccataccgcg
aaaggttttgcgccattcgatggtgtccgggatctcgacgctctcccttatgcgactcctgcattaggaagcagcccagtagtaggttga
ggccgttgagcaccgccgccgcaaggaatggtgcatgcaaggagatggcgcccaacagtcccccggccacggggcctgccacc
atacccacgccgaaacaagcgctcatgagcccgaagtggcgagcccgatcttccccatcggtgatgtcggcgatataggcgccag
caaccgcacctgtggcgccggtgatgccggccacgatgcgtccggcgtagaggatcgagatctcgatcccgcgaaattaatacgac
tcactataggggaattgtgagcggataacaattcccctctagaaataattttgtttaactttaagaaggagatatacatatgaaatacctg
ctgccgaccgctgctgctggtctgctgctcctcgctgcccagccggcgatggcccaagtccaattacaagagtccggcggcggacttg
tccagcctgggggatcattgcgcctgtcgtgtgcggcgtcgggatttgtatttgaaaatagtgccatggcctggtatcgtcaggcgcctgg
gcgtgagcgtgagctgattgctgtgattggaactacattcattcgtttggctgaatctgtgcgtgggcgttttacgatcagccgcgataatg
cacgttctacggtttatttgcaaatgaataatttacgtcctgaagacacagcggtttactactgttcaaaatcgggagcgtattggggaca
ggggacacaagtaactgtgtcatctggcgggctgcccgagacgggcggccaccaccaccaccaccactgagatccggctgctaac
aaagcccgaaaggaagctgagttggctgctgccaccgctgagcaataactagcataaccccttggggcctctaaacgggtcttgag
gggttttttgctgaaaggaggaactatatccggat

<u>Nb$_{alfa}$</u>

nagcgcccaatacgcaaaccgcctctccccgcgcgttggccgattcattaatgcagctggcacgacaggtttcccgactggaaagcg
ggcagtgagcgcaacgcaattaatgtgagttagctcactcattaggcaccccaggctttacactttatgcttccggctcgtatgttgtgtgg
aattgtgagcggataacaatttcacacaggaaacagctatgaccatgattacgccaagcttgcatgcaaattctatttcaaggagaca

gtcataatgaaatacctattgcctacggcagccgctggattgttattactcgcggcccagccggccatggctgaagtacagcttcagga
atccggtggcggtctggtacagccgggtggctccctgcgtctgagttgtaccgctagcggtgttaccatttctgccttaaacgccatggcg
atgggctggtatcgccaagcgccgggagaacgtcgcgtcatggtagcggccgtatcggagcgtggcaatgcgatgtaccgtgaaag
cgtacaagggcgctttacggtaacgcgcgacttcacgaacaaaatggtaagcttacaaatggataatctgaagccggaagacacg
gcagtgtattactgtcacgtattggaggatcgcgttgactctttccatgattattgggggcaaggtacacaggtgacggtatcctcagtctc
ctcaggaggactgccggaaaccggcggccaccaccatcaccatcactaatagaattcactggccgtcgttttacaacgtcgtgactg
ggaaaaccctggcgttacccaacttaatcgccttgcagcacatccccctttcgccagctggcgtaatagcgaagaggcccgcaccga
tcgcccttcccaacagttgcgcagcctgaatggcgaatggcgcctgatgcggtattttctccttacgcatctgtgcggtatttcacaccgc
atacgtcaaagcaaccatagtacgcgccctgtagcggcgcattaagcgcggcgggtgtggtggttacgcgcagcgtgaccgctaca
cttgccagcgccctagcgcccgctcctttcgctttcttcccttcctttctcgccacgttcgccggctttccccgtcaagctctaaatcgggggc
tccctttagggttccgatttagtgctttacggcacctcgaccccaaaaaacttgatttgggtgatggttcacgtagtgggccatcgccctga
tagacggtttttcgccctttgacgttggagtccacgttctttaatagtggactcttgttccaaactggaacaacactcaaccctatctcgggct
attcttttgatttataagggattttgccgatttcggcctattggttaaaaaatgagctgatttaacaaaaatttaacgcgaattttaacaaaata
ttaacgtttacaattttatggtgcactctcagtacaatctgctctgatgccgcatagttaagccagccccgacacccgccaacacccgctg
acgcgccctgacgggcttgtctgctcccggcatccgcttacagacaagctgtgaccgtctccgggagctgcatgtgtcagaggttttca
ccgtcatcaccgaaacgcgcgagacgaaagggcctcgtgatacgcctatttttataggttaatgtcatgataataatggtttcttagacgt
caggtggcacttttcggggaaatgtgcgcggaacccctatttgtttatttttctaaatacattcaaatatgtatccgctcatgagacaataac
cctgataaatgcttcaataatattgaaaaaggaagagtatgagtattcaacatttccgtgtcgcccttattcccttttttgcggcattttgccttc
ctgtttttgctcacccagaaacgctggtgaaagtaaaagatgctgaagatcagttgggtgcacgagtgggttacatcgaactggatctc
aacagcggtaagatccttgagagttttcgccccgaagaacgttttccaatgatgagcacttttaaagttctgctatgtggcgcggtattatc
ccgtattgacgccgggcaagagcaactcggtcgccgcatacactattctcagaatgacttggttgagtactcaccagtcacagaaaa
gcatcttacggatggcatgacagtaagagaattatgcagtgctgccataaccatgagtgataacactgcggccaacttacttctgacaa
cgatcggaggaccgaaggagctaaccgcttttttgcacaacatgggggatcatgtaactcgccttgatcgttgggaaccggagctgaa
tgaagccataccaaacgacgagcgtgacaccacgatgcctgtagcaatggcaacaacgttgcgcaaactattaactggcgaacta
cttactctagcttcccggcaacaattaatagactggatggaggcggataaagttgcaggaccacttctgcgctcggcccttccggctgg
ctggtttattgctgataaatctggagccggtgagcgtgggtctcgcggtatcattgcagcactggggccagatggtaagccctcccgtat
cgtagttatctacacgacggggagtcaggcaactatggatgaacgaaatagacagatcgctgagataggtgcctcactgattaagca
ttggtaactgtcagaccaagtttactcatatatactttagattgatttaaaacttcatttttaatttaaaaggatctaggtgaagatcctttttgat
aatctcatgaccaaaatcccttaacgtgagttttcgttccactgagcgtcagaccccgtagaaaagatcaaaggatcttcttgagatcctt
tttttctgcgcgtaatctgctgcttgcaaacaaaaaaaccaccgctaccagcggtggtttgtttgccggatcaagagctaccaactctttttc
cgaaggtaactggcttcagcagagcgcagataccaaatactgtccttctagtgtagccgtagttaggccaccacttcaagaactctgta
gcaccgcctacatacctcgctctgctaatcctgttaccagtggctgctgccagtggcgataagtcgtgtcttaccgggttggactcaaga
cgatagttaccggataaggcgcagcggtcgggctgaacggggggttcgtgcacacagcccagcttggagcgaacgacctacacc
gaactgagatacctacagcgtgagctatgagaaagcgccacgcttcccgaagggagaaaggcggacaggtatccggtaagcggc
agggtcggaacaggagagcgcacgagggagcttccagggggaaacgcctggtatctttatagtcctgtcgggtttcgccacctctga
cttgagcgtcgatttttgtgatgctcgtcaggggggcggagcctatggaaaaacgccagcaacgcggcctttttacggttcctggcctttt
gctggccttttgctcacatgttctttcctgcgttatcccctgattctgtggataaccgtattaccgcctttgagtgagctgataccgctcgccgc
agccgaacgaccgagcgcagcgagtcagtgagcgaggaagcggaag

NK1R sequence

GPSRLEEELRRRLTEPGQADQEAKELARQISGPDRVRAVSHWSSMDNVLPVDSDLSPNISTNT
SEPNQFVQPAWQIVLWAAAYTVIVVTSVVGNVVVMWIILAHKRMRTVTNYFLVNLAFAEASMAA
FNTVVNFTYAVHNEWYYGLFYCKFHNFFPIAAVFASIYSMTAVAFDRYMAIIHPLQPRLSATATK

VVICVIWVLALLLAFPQGYYSTTETMPSRVVCMIEWPEHPNKIYEKVYHICVTVLIYFLPLLVIGYA
YTVVGITLWASEIPGDSSDRYHEQVSAKRKVVKMMIVVVCTFAICWLPFHIFFLLPYINPDLYLKK
FIQQVYLAIMWLAMSSTMYNPIIYCCLNDRFRLGFKHAFRCCPFISAGDYEGLEMKSTRYLQTQ
GSVYKVSRLETTISTVVGAHEEEPEDGPKATPSSLDLTSNCSSRSDSKTMTESFSFSSNVLSDY
KDDDDK

Highlighted alfa, 6e and BC_2 tags

Nb sequences

Nb_{6e}: QVQLQESGGG LVQPGGSLRL SCAASGFVFE NSAMAWYRQA PGKERELIAV IGTTFIKLAE SVKGRFTISR DNAKSTVYLQ MNNLKPEDTA VYYCSKSGAY WGQGTQVTVS SGGLPETGHH HHHH

Nb_{alfa}: EVQLQESGGG LVQPGGSLRL SCTASGVTIS ALNAMAMGWY RQAPGERRVM VAAVSERGNA MYRESVQGRF TVTRDFTNKM VSLQMDNLKP EDTAVYYCHV LEDRVDSFHD YWGQGTQVTV SSGGLPETGG HHHHHH

Nb_{BC2}: QVQLVESGGG LVQPGGSLTL SCTASGFTLD HYDIGWFRQA PGKEREGVSC INNSDDDTYY ADSVKGRFTI FMNNAKDTVY LQMNSLKPED TAIYYCAEAR GCKRGRYEYD FWGQGTQVTV SSGGLPETGG HHHHHH

Nb_{GFP}: QVQLQESGGA LVQPGGSLRL SCAASGFPVN RYSMRWYRQA PGKEREWVAG MSSAGDRSSY EDSVKGRFTI SRDDARNTVY LQMNSLKPED TAVYYCNVNV GFEYWGQGTQ VTVSSGGLPE TGGHHHHHH

Table S1. Nanobody-epitope tag pairs

Tag	Sequence
6e	QADQEAKELARQIS
Alfa	SRLEEELRRRLTE
BC_2	PDRVRAVSHWSS

Table S2. List of Nb conjugates calculated and observed masses

Nb conjugates	calculated mass after sortagging	observed mass
Nb_{alfa}-LPTGG-His_6	-	15207
Nb_{6e}-LPTGG-His_6	-	13476
Nb_{BC2}-LPTGG-His_6	-	14993
Nb_{GFP}-LPTGG-His_6	-	14233
Nb_{alfa}-NKA	15556	15555
Nb_{6e}-NKA	13825	13828
Nb_{BC2}-NKA	15342	15338
Nb_{GFP}-NKA	14582	14583
Nb_{alfa}-SP_{6-11}	15164	15165
Nb_{6e}-SP_{6-11}	13433	13436
Nb_{BC2}-SP_{6-11}	14950	14951
Nb_{GFP}-SP_{6-11}	14190	14189
